# Mapping of Agricultural Subsurface Drainage Systems Using Unmanned Aerial Vehicle Imagery and Ground Penetrating Radar †

**DOI:** 10.3390/s21082800

**Published:** 2021-04-15

**Authors:** Triven Koganti, Ehsan Ghane, Luis Rene Martinez, Bo V. Iversen, Barry J. Allred

**Affiliations:** 1 Department of Agroecology, Aarhus University, Blichers Allé 20, 8830 Tjele, Denmark; bo.v.iversen@agro.au.dk; 2 Department of Biosystems and Agricultural Engineering, Michigan State University, East Lansing, MI 48824, USA; ghane@msu.edu; 3 USDA/ARS Soil Drainage Research Unit, 590 Woody Hayes Drive, Columbus, OH 43210, USA; luis.martinez@usda.gov (L.R.M.); barry.allred@ars.usda.gov (B.J.A.)

**Keywords:** tile drainage, unmanned aerial vehicles, visible-color imagery, multispectral imagery, thermal infrared imagery, ground penetrating radar, non-destructive techniques

## Abstract

Agricultural subsurface drainage systems are commonly installed on farmland to remove the excess water from poorly drained soils. Conventional methods for drainage mapping such as tile probes and trenching equipment are laborious, cause pipe damage, and are often inefficient to apply at large spatial scales. Knowledge of locations of an existing drainage network is crucial to understand the increased leaching and offsite release of drainage discharge and to retrofit the new drain lines within the existing drainage system. Recent technological developments in non-destructive techniques might provide a potential alternative solution. The objective of this study was to determine the suitability of unmanned aerial vehicle (UAV) imagery collected using three different cameras (visible-color, multispectral, and thermal infrared) and ground penetrating radar (GPR) for subsurface drainage mapping. Both the techniques are complementary in terms of their usage, applicability, and the properties they measure and were applied at four different sites in the Midwest USA. At Site-1, both the UAV imagery and GPR were equally successful across the entire field, while at Site-2, the UAV imagery was successful in one section of the field, and GPR proved to be useful in the other section where the UAV imagery failed to capture the drainage pipes’ location. At Site-3, less to no success was observed in finding the drain lines using UAV imagery captured on bare ground conditions, whereas good success was achieved using GPR. Conversely, at Site-4, the UAV imagery was successful and GPR failed to capture the drainage pipes’ location. Although UAV imagery seems to be an attractive solution for mapping agricultural subsurface drainage systems as it is cost-effective and can cover large field areas, the results suggest the usefulness of GPR to complement the former as both a mapping and validation technique. Hence, this case study compares and contrasts the suitability of both the methods, provides guidance on the optimal survey timing, and recommends their combined usage given both the technologies are available to deploy for drainage mapping purposes.

## 1. Introduction

### 1.1. Research Rationale

Subsurface drainage systems are installed in agricultural areas to remove excess water and convert poorly drained soils into productive cropland. Some of the most productive agricultural regions in the world are a result of subsurface drainage practices [1]. Subsurface drainage provides many agronomic, economic, and environmental benefits by lowering the water table, enhancing optimal conditions for proper aeration of the plant roots and improving trafficability for timeliness of field operations, thereby increasing crop yields [2,3]. However, drain lines also shorten pathways for solute transport, causing increased leaching and offsite release of nutrients, pathogens, and pesticides from the agricultural areas, in turn increasing the potential risk for eutrophication and contamination of surface water bodies [4,5,6,7]. Hence, knowledge of subsurface drainage system locations is important for the understanding of the local hydrology and solute dynamics for consequent planning of mitigation strategies such as constructed wetlands, saturated buffers, denitrifying bioreactors, and phosphate filters [8,9,10,11,12,13].

**Figure 1:**
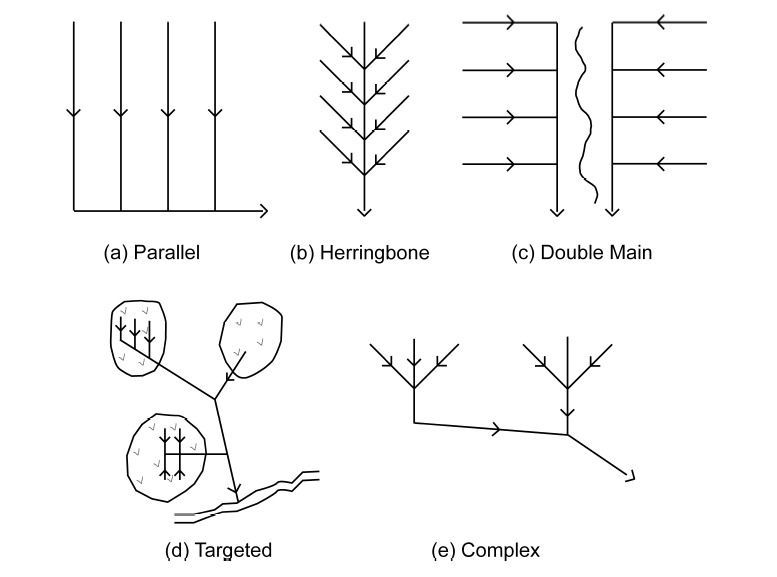
Commonly used subsurface drainage system patterns (modified from [24,25]).

In addition, the installation of a new set of drain lines to enhance soil water removal efficiency or for sub-irrigation requires accurate knowledge of the existing drainage system as the new drain lines are typically installed between the old drain lines [14,15]. This is also true for damaged drainage pipes as farmers need their precise location before initiating repairs [16]. Recent reviews by Valipour et al. [17] and Yannopoulos et al. [18] provide a comprehensive overview of the evolution of agricultural drainage and materials and methods used from antiquity to the present. Old drainage pipes are made up of clay/ceramic tiles, and hence “subsurface drainage systems” are still commonly referred to as “tile drainage systems”. However, since the 1980s, corrugated high-density polyethylene and polyvinyl chloride pipes are considered as a standard for subsurface drainage installations [19,20]. The drainage systems are typically installed at a depth of 0.6–1.5 m, spaced 8–30 m apart, and the pipe diameters range between 50 and 200 mm [21,22]. The drainage design depends on the inventory available, soil types, and drainage catchment size. Moreover, as the old drainage pipes become less efficient with time due to clogging by sediment deposition, they are often left in place, even if non-functional, as it is neither economical nor practical to remove them [23]. Despite the importance for environmental risk assessment and efficient agricultural land management, the location of the drainage pipe installations is often poorly documented or non-existent, requiring the need for extensive mapping campaigns. Figure 1 shows the typical patterns followed for the installation of subsurface drainage systems.

The traditional methods of drainage mapping involve the use of tile probes and trenching equipment. While the tile probes are time-consuming, tedious, and hard to employ across large spatial scales, the use of trenching equipment, though effective, is extremely invasive and severs the drainage pipes, thus requiring costly repairs [26]. Non-destructive techniques, both proximal and remote, commonly used for soil and crop sensing studies may potentially provide an alternative solution. In proximal sensing techniques, previous research demonstrated considerable success using time-domain ground penetrating radar (GPR) in finding the drainage pipes [14,15,16,27,28,29]. Although non-invasive and effective in many circumstances, the necessity to perform the survey along multiple parallel transects or according to a grid to obtain complete coverage of the field area can sometimes be impractical and challenging. More recently, Allred et al. [26] proposed using spiral, serpentine segments incorporated into a few random, parallel, and perpendicular transects as an alternative, and Koganti et al. [30] and Koganti et al. [31] used a stepped-frequency continuous wave 3D-GPR system with a wide frequency bandwidth and wide antenna array swathe for effective coverage of the 3D space to confirm the presence of a drain line and to ascertain its orientation.

In relation to remote sensing techniques, most of the previous studies analyzed satellite imagery or aerial imagery from manned aircraft or helicopters to study subsurface drainage systems [32,33,34,35,36,37,38]. While the coarser resolution of satellite imagery limited its capability to determine the areas that are potentially drained from undrained, high-resolution aerial photos showed promise in finding the precise location in some instances, although collecting such imagery could turn out to be expensive. Recent technological advancements in remote sensing enabled unmanned aerial vehicles (UAVs) and their compatible cameras—visible-color (VIS-C), multispectral (MS), and thermal infrared (TIR)—to become affordable. A few recent studies [39,40,41,42,43,44,45,46] explored the potential of UAV imagery for subsurface drainage mapping. However, more work is warranted in this direction as the research is still in a beginning stage, with ideal conditions based on soil type, crop residue, tillage practice, ground wetness level, and prior rainfall event for carrying out the UAV surveys for subsurface drainage mapping not yet fully understood. Consequently, given suitable conditions, both GPR and UAV imagery have proven useful for drainage mapping purposes.

### 1.2. Justification Supporting the Use of GPR and UAV Imagery in Combination

GPR works in a frequency range of 10 MHz to 1 GHz and consists of transmitter and receiver antennas. The transmitter antenna transmits electromagnetic (EM) energy into the ground and a receiver antenna records the earth’s impulse response, i.e., the reflected and scattered energy. Two electrical properties—relative dielectric permittivity (RDP) and electrical conductivity (EC)—mainly control the GPR wave propagation in the subsurface as the magnetic permeability is generally assumed a constant [47]. The degree of contrast in RDP encountered at the boundary between two different materials controls the strength of the reflection and the soil EC determines the GPR signal attenuation and penetration depth (PD) [48,49]. The reflection coefficient is positive and the polarity of the reflected wave is the same as the incident wave when a propagating GPR wave encounters a high RDP medium, whereas a polarity reversal occurs in case the medium encountered is of relatively low RDP [14,48]. In addition, RDP also controls the GPR wave velocity in the subsurface and the time delay between the transmission and detection (two-way travel time) is proportional to the depth of the contrast.

In the case of drainage pipe mapping, the detectability arises because of the contrast in RDP between the material inside the drainage pipe (air/water) and the material surrounding the drainage pipe (soil). The material of the pipe itself (clay/ceramic/polyvinyl chloride/polyethylene) has no effect on the GPR drainage pipe response [14,16,50]. Moreover, as the GPR signal propagates into the subsurface as an elongated cone of energy [51], it “sees” buried features both in front of it and behind it. Hence, when GPR is moved on the top of a point size object (rocks, cavities, etc.), the latter produces a hyperbolic signature on the GPR vertical profile. Lastly, the choice of antenna bandwidth is an important consideration while employing GPR. A wider frequency bandwidth enables better horizontal and vertical resolutions. However, in lossy dispersive media (such as soils), the improvement in resolution is at a compromise of a decrease in the signal PD as the high-frequency EM waves attenuate relatively quickly when compared to lower frequencies [49,52,53,54]. This constraint is often referred to as the “range–resolution” trade-off of the GPR technology and a careful choice of the antenna bandwidth is necessary depending on the investigation purpose (i.e., the desired resolution and signal PD).

Drainage pipes at their usual depth of installation (0.6–1.5 m) can be regarded as a point size object and therefore show up as hyperbolas in vertical profiles obtained along a perpendicular traverse relative to the drainage pipe orientation. If the GPR transect is over the top and along the trend of a drain line, it will show up as a banded linear feature in the vertical profile [14,26,31]. The advantages of using GPR for drainage pipe mapping are that it provides depth information and can confirm the signature is actually caused by a drainage pipe. The limitation is the limited spatial coverage as it is expensive to collect and interpret the data over large farm field areas. In addition, the agricultural soils that have a high EC might severely limit the penetration of the GPR signal, thereby restricting the drainage pipes’ detectability [31]. In this relation, GPR surveys are generally recommended to be carried out two to three days after a rainfall event, once the soil profile reaches “field capacity” conditions or anytime thereafter, i.e., when the water table recedes until or beyond the drain line depth. Further, bare ground conditions are preferred for easier mobility and enhanced coupling of the GPR antennas with the ground [14,55].

The detectability of drain line signatures in UAV imagery is possible due to the greater water removal and soil drying directly above a drain line after a significant rainfall event in comparison to the soil between the drain lines [33,37,43]. Consequently, given bare ground conditions, lighter shaded dry soil surface features (i.e., increased reflected radiation) that are linear may be representative of drain lines as the dry soil surfaces reflect more visible (VIS) and near-infrared (NIR) EM radiation than wet soil surfaces [56,57]. This makes VIS-C and MS (green, red, red edge, and NIR wavelength bands) cameras a suitable choice for subsurface drainage mapping [40,42,44,57]. In addition, soil water content variation can result in emitted TIR radiation differences explained by the difference in thermal inertia between dry and wet soils due to the high specific heat capacity and low thermal conductivity of water resulting in a temperature difference [58,59,60]. Additionally, there might be emissivity differences between dry and wet soils, subsequently making TIR cameras a potential tool for drainage pipe detection [39,40,43,46,61]. Therefore, earlier studies recommend capturing the aerial imagery outside the growing season (with bare ground conditions) two to three days after a significant (2.5 cm and greater) rainfall event as the optimal timing for drainage mapping purposes [33,34].

Furthermore, early into the growing season, the crops tend to establish first directly above the drain lines and are in better health compared to in between the drain lines as optimal soil–water–air conditions are established for proper aeration [35,40,45]. This results in possible drainage pipe locations showing up as distinct linear features on the VIS-C imagery and index maps indicative of crop establishment such as NDVI (Normalized Difference Vegetation Index) and NDRE (Normalized Difference Red Edge Index) generated from the MS imagery. Additionally, they can become visible on the TIR imagery which can also detect the spatial variation in crop health or stress [62,63,64]. The advantages with the use of UAV-based sensory technology are that it is flexible to schedule a survey and inexpensive to cover large farm field areas in a limited time. The limitations are the inability of the UAV imagery to provide the depth information of the drainage pipes unlike GPR, their dependence on timing concerning soil wetness and site surface conditions, and the lack of distinction between the signature caused by drainage pipes from that caused due to field operations such as compacted soil wheel tracks from either harvest, tillage, fertilizer, and planting equipment; stalks and chaff expelled/deposited behind combine harvesters; or wide bands of crop residue [40]. However, it should be noted that guidelines have been developed by Allred et al. [40] to easily distinguish drain line responses from those due to farm field operations.

Therefore, on the one hand, we have GPR that responds to subsurface variation in soil electrical properties, provides depth information, and has showed significant success in drainage pipe detection. At the same time, it is expensive to employ across large farm field areas and has limited PD in high-EC soils. On the other hand, we have the newly emerging UAV-based sensory technology which measures the surface variation in soil and plant properties, is more feasible, and has showed promise for drainage pipe mapping across large field areas. However, it comes with its own set of drawbacks such as the inability to detect or demarcate the drain lines’ location under certain conditions. Given the complementary nature of their usage, applicability, and the information they provide concerning drainage pipe mapping, further investigation is needed regarding their combined use.

### 1.3. Research Focus

In this study, we evaluate the potential of UAV imagery in combination with GPR across four study sites in the Midwest USA for mapping agricultural subsurface drainage systems. The UAV imagery and GPR surveys were performed flexibly when an opportunity arose and without adhering to any specific criteria concerning the prior rainfall and site surface conditions. The UAV imagery data were collected using three different cameras (VIS-C, MS, and TIR) and the GPR data were collected across a limited spatial extent, preferably in the direction perpendicular to the expected drain line orientation or in a random fashion when the orientation was unknown, by using parallel, spiral, and serpentine transects. The hypotheses tested were as follows: (1) the UAV imagery might not be able to capture the drain line signature on all the different soil types due to non-existent ideal conditions concerning prior rainfall, ground wetness level, crop residue, time of day the imagery was captured, etc., (2) the drainage pipe response can vary between different bandwidths of imagery, and (3) the use of GPR in combination is useful at least on a limited spatial extent as it acts as validation by confirming the drainage pipe signature depicted in the UAV imagery, providing information on drainage pipes’ depth and possibly ascertaining whether the farm field is subsurface drained or not. Overall, the case study presented here explores the suitability of both technologies, comparing and contrasting their abilities, and provides insight into their complementary use. Further, an attempt was made to develop guidelines on the optimal survey timing concerning prior rainfall (i.e., soil wetness) and site surface (i.e., bare ground/crop cover) conditions for employing these sensors to derive maximal success in subsurface drainage mapping.

## 2. Materials and Methods

### 2.1. Study Sites

**Figure 2:**
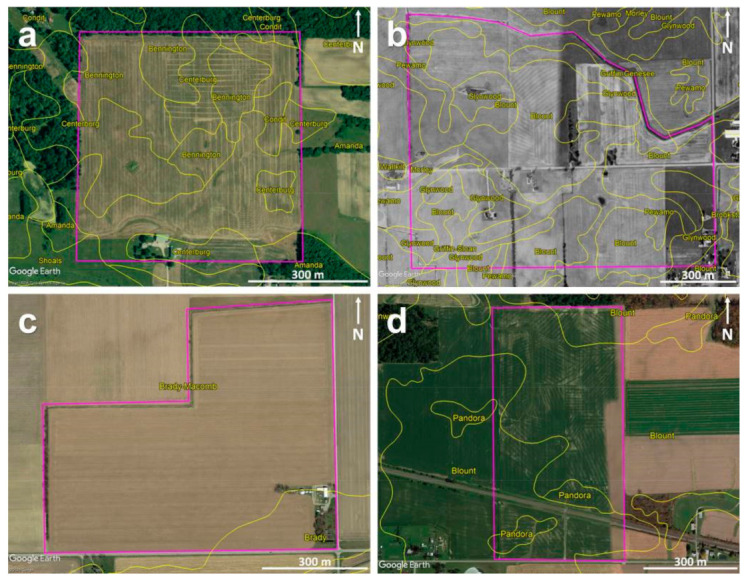
Google Earth aerial images of the agricultural sites under investigation overlaid with soil maps (in yellow) from SoilWeb-Earth: (**a**) Site-1, (**b**) Site-2, (**c**) Site-3, and (**d**) Site-4. Purple lines mark the site boundaries.

**Figure 3:**
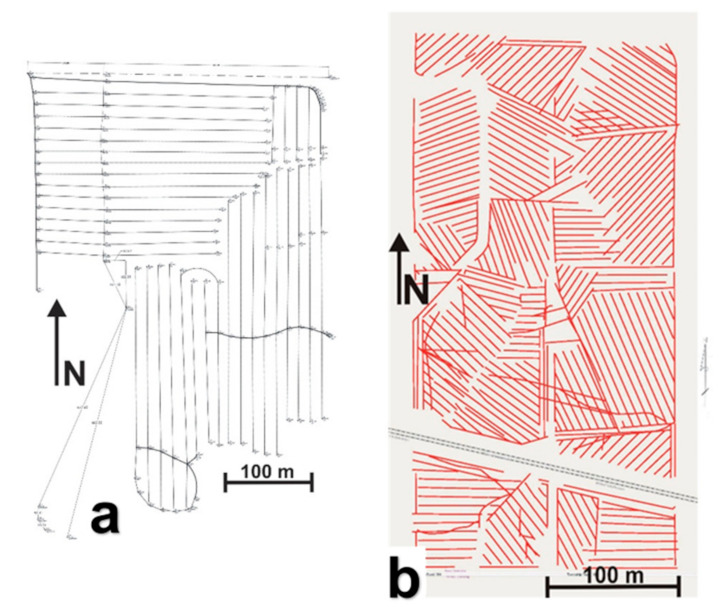
Existing site drainage maps: (**a**) Site-1 and (**b**) Site-4.

Two sites in Ohio (OH) and two sites in Michigan (MI), all within the Midwest USA, were visited as a part of this study. Figure 2 shows the aerial images of the four sites obtained via Google Earth (Google LLC., Mountain View, CA, USA) overlaid upon with the soil maps from SoilWeb-Earth [65]. At Site-1, the aerial image captured on 4 June 2009, by the US Department of Agriculture—Farm Service Agency, was used, as it clearly revealed most of the subsurface drainage system locations in the eastern part of the site installed in August 2008. Additionally, it closely aligns with the drainage map secured from the contractor (Figure 3a). Similarly, at Site-2, the mosaic created by aerial imagery captured between 22 April 1998 and 30 March 1999, by the US Geological Survey, was used, as this revealed at least some of the drainage pipe locations at this site. At Site-3, the most recent aerial imagery captured on 29 April 2018 was used as none of the historical imagery showed any indications of the drainage installations. Again, at Site-4, the mosaic created by aerial imagery captured between 25 October 2015 and 11 July 2018 was used as this revealed most of the drain line locations as differences in the crop development. A detailed description of the soil types, surface conditions, and cumulative rainfall three days prior to the surveys at each site is provided below and summarized in Table 1.

Site-1, OH:

Site-1 is located in Morrow County of central OH. According to SoilWeb-Earth [65], the soils at this site include Amanda silt loam (6–18% slopes—fine-loamy, mixed, mesic Typic Hapludalfs), Bennington silt loam (0–6% slopes—fine, illitic, mesic Aeric Epiaqualfs), Centerburg silt loam (2–6% slopes—fine-loamy, mixed, active, mesic Aquic Hapludalfs), and Condit silt loam (0–1% slope—fine, illitic, mesic Typic Epiaqualfs). The area of interest is around 33 ha (Figure 2a), and the maximum elevation difference, from the lowest to highest points, is approximately 10 m [39]. This site was intensively investigated over the last few years for subsurface drainage pipe mapping purposes [39,40]. On May 6, 2019, during the recent UAV flights, the site conditions were extensive bare ground throughout the entire field with soybean stubble on the east side and corn stubble on the west side. The GPR surveys were performed between 2 and 6 May 2019, simultaneously with the recent UAV flights.

Site-2, MI:

Site-2 is located in Lenawee County of MI. The soils at this site include Blount loam (0–6% slopes—fine, illitic, mesic Aeric Epiaqualfs), Glynwood loam (2–6% slopes—fine, illitic, mesic Aquic Hapludalfs), Griffin and Genesee loams (0–3% slopes—fine-loamy, mixed, nonacid, mesic Aeric Fluvaquents and fine-loamy, mixed, superactive, mesic Fluventic Eutrudepts, respectively), Pewamo clay and mucky clay loam (0–3% slopes—fine, mixed, active, mesic Typic Argiaquolls), Griffin and Sloan sandy loams (0–3% slopes—fine-loamy, mixed, nonacid, mesic Aeric Fluvaquents and fine-loamy, mixed, superactive, mesic Fluvaquentic Endoaquolls, respectively), and Morley loam (6–12% slopes—fine, illitic, mesic Typic Hapludalfs). The area of interest is around 100 ha (Figure 2b). The site conditions were limited bare ground to the north side of the road with an early-stage soybean crop development and extensive bare ground to the south side of the road with an early-stage corn crop during the UAV flights carried out on 7 May 2018. During the GPR survey and the recent flights carried out on 21 May 2019, the site conditions were an established cereal ryegrass cover crop across the entire field.

Site-3, MI:

Site-3 is located in Lenawee County of MI. The soils at this site are mostly Brady and Macomb loams (0–3% slopes—coarse-loamy, mixed, active, mesic Aquollic Hapludalfs and fine-loamy, mixed, semiactive, mesic Aquollic Hapludalfs, respectively) and Brady sandy loam (0–3% slopes—coarse-loamy, mixed, active, mesic Aquollic Hapludalfs). The area of interest is around 45 ha (Figure 2c), and the site conditions were extensive bare ground throughout the field during the UAV surveys carried out on 7 May 2018, and established soybean crop during the GPR and UAV surveys performed on 12 July 2018. Here, a more extensive set of GPR surveys was conducted on 10 December 2019, on bare soil.

Site-4, OH:

Site-4 is located in Seneca County of OH. The soils here are mainly Blount silt loam (2–4% slopes—fine, illitic, mesic Aeric Epiaqualfs) and Pandora silt loam (fine, mixed, mesic Typic Ochraqualfs). The area of interest is around 33 ha (Figure 2d), and the site conditions were extensive bare ground with a substantial amount of soybean residue during the UAV and GPR surveys carried out on 21 June 2019.

**Table 1:** Summary of the study sites’ location, soil types, site survey conditions, dates of the unmanned aerial vehicle (UAV) imagery and ground penetrating radar (GPR) surveys, and cumulative rainfall values (in brackets) three days prior to the surveys.

Site Name	Soil Types *	Site Conditions	Date of the UAV Surveys and 3 Days’ Prior Rainfall ^#^ (mm)	Date of the GPR Surveys and 3 Days’ Prior Rainfall ^#^ (mm)
Site-1, OH	Silt loam	Bare ground with corn stubble to the west side and soybean stubble to the east side	6 May 2019 (18.5)	2–6 May 2019 (18.5)
Site 2, MI	Sandy loam, loam, clay loam	Limited and extensive bare ground, respectively, to the north and south of the road with an early-stage soybean and corn crop development (7 May 2018); established cereal ryegrass cover crop (21 May 2019)	7 May 2018 (1.5); 21 May 2019 (19.9)	21 May 2019 (19.9)
Site 3, MI	Sandy loam, loam	Extensive bare ground (7 May 2018); established soybean crop (12 July 2018); and extensive bare ground (10 December 2019)	7 May 2018 (3.1); 12 July 2018 (0)	12 July 2018 (0); 10 December 2019 (5.6)
Site 4, OH	Silt loam	Substantial soybean residue on extensive bare ground	21 June 2019 (44.7)	21 June 2019 (44.7)

* Soil type details obtained from SoilWeb-Earth [65]. # Rainfall data obtained from closest NOAA—National Weather Service station with complete daily rainfall record [66].

Figure 3 shows the pre-existing drainage maps from Sites-1 and -4. No pre-existing maps are available at Sites-2 and -3. The contractor’s drainage map for the installations made in August 2008, at Site-1 to the eastern part of the field, shows a complex drainage pattern with both east–west- and north–south-trending drain lines (Figure 3a). These drain lines were clearly visible in the Google Earth imagery (Figure 2a). In the northwestern part of the field, there is a known set of north–south-trending drain lines made of corrugated plastic tubing installed in 1986 [39]; however, no prior construction maps are available for these older installations. To the southwestern part, the drainage pattern is unknown, though a few random drain lines installed in the early 1900s may be present here and elsewhere in the field. At Site-2, as can be seen in the Google Earth imagery (Figure 2b), the drainage pattern is a complex herringbone system with the drain lines trending in the northeast–southwest orientation in the northwestern part. No drainage signature was observed in the central part of the field north of the road. To the central part of the field south of the road, the drain lines mostly trend in the northeast–southwest and northwest–southeast orientations, though only visible to a lesser extent. At Site-3, the drainage pattern is unknown due to the lack of pre-existing drainage maps or visible drainage signatures in the historical Google Earth imagery. At Site-4, the drainage pattern is extremely complex with the drain lines trending in north–south, east–west, northwest–southeast, and northeast–southwest orientations (Figure 3b).

### 2.2. UAV Equipment, Survey, and Data Processing

#### 2.2.1. Equipment

**Figure 4:**
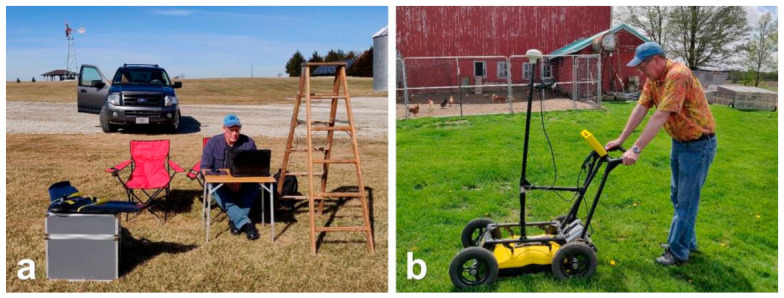
Equipment used in the investigation: (**a**) fixed-wing UAV, ladder with ground modem antenna, and computer, (**b**) SmartCart Noggin GPR system (250 MHz antennas).

A fixed-wing UAV, eBee Plus (senseFly SA, Cheseaux-sur-Lausanne, Switzerland; Figure 4a), with real-time kinematic (RTK) Global Navigation Satellite System (GNSS) functionality, was used in this study. The UAV could accommodate only one camera payload at a time, so separate flights were carried out using the VIS-C (S.O.D.A.), MS (Sequoia), and TIR (thermoMAP) cameras. A connection to the UAV was established by using a computer and ground modem antenna, and field internet access was obtained through an internal cellular modem in the computer. The senseFly SA eMotion3 software was used to control the flight plan and to manage the data collected during each survey. During the flight, the UAV typically achieved speeds of 37–65 km/h (20–35 kn).

The senseFly SA S.O.D.A. (Sensor Optimized for Drone Applications) is a 20 Mpx camera with a 28 mm focal lens and was used to collect high-resolution (2.8 cm/px) panchromatic VIS-C (400–700 nm wavelengths) photos. The UAV, when configured with the S.O.D.A. camera, had RTK/GNSS functionality and provided survey-grade positional accuracy (0.7 cm) for the VIS-C imagery obtained. To use RTK/GNSS functionality with the UAV S.O.D.A. configuration, onsite connection via the internet was achieved with state (OH and MI) Department of Transportation virtual reference station networks. A lateral photo overlap of 70% between the adjacent flight lines and a longitudinal photo overlap of 60% along the flight line was maintained while flying with the S.O.D.A. camera.

The Sequoia (Parrot SA, Paris, France) MS camera captured 16 Mpx photos in VIS-C and 1.2 Mpx photos in four narrow bands of green (530–570 nm), red (640–680 nm), red edge (730–740 nm), and NIR (770–810 nm) wavelengths, respectively. The UAV, when configured with the Sequoia camera, did not have RTK/GNSS functionality. Hence, the positional accuracy was approximately 4 m for the imagery obtained. A lateral photo overlap of 60% and a longitudinal overlap of 80% were maintained while flying with the Sequoia camera. The senseFly SA thermoMAP TIR camera captured 0.3 Mpx photos in a wavelength range of 8.5–11.5 μm. Again, the UAV, when configured with the thermoMAP camera, did not have RTK/GNSS functionality, and the positional accuracy of the photos was approximately 4 m. A lateral photo overlap of 70% and a longitudinal photo overlap of 90% were maintained while flying with the thermoMAP camera. It is worth a mention that for the UAV flights carried out at a similar height, both the spatial and spectral resolutions of a TIR camera are, in general, coarser when compared to the VIS-C and MS cameras (see Table 2). This is because a TIR camera records the energy emitted by a surface/object (with an absolute temperature above 0 K), whereas the VIS-C and MS cameras register the reflected energy when a surface/object is illuminated by the solar irradiance. Since the thermal emissions from the earth’s surface (typically at about 300 K temperature) are significantly less when compared to the reflected solar energy, a sensor measuring these emissions needs to scan a wider area over a broad wavelength bandwidth to register a noticeable amount of energy [67].

Cloud-connected, high-precision AeroPoints (Propeller Aerobotics Pty. Ltd., Surry Hills, NSW, Australia) were used as ground control points (GCPs) to confirm the positional accuracy of the UAV S.O.D.A. surveys and to greatly improve the positional accuracy of the UAV Sequoia surveys. For the TIR imagery, the AeroPoints did not show up well, and hence a 61-cm (24 in.)-diameter aluminum pizza pan was placed next to each AeroPoint in the field. The aluminum pizza pans showed up well on both S.O.D.A. and thermoMap images. Consequently, the S.O.D.A. survey was used to obtain accurate positional coordinates for the pizza pans, thereby allowing the pizza pans to be employed as precision GCPs for the thermoMap survey. This approach greatly improved the locational accuracy of the thermoMap imagery.

#### 2.2.2. Survey Information

At Site-1, the UAV flights were performed on 6 May 2019, using all three camera payloads. At Sites-2 and -3, the UAV flights were initially performed on 7 May 2018, with two cameras (S.O.D.A. and thermoMAP), and the recent surveys with all three cameras were performed, respectively, on 21 May 2019, and 12 July 2018. At Site-4, two sets of UAV surveys at different times during the day were performed on 21 June 2019, with all three cameras. The flights were carried out at approximately 117 m height above the ground as 122 m was the maximum height allowed by the U.S. Federal Aviation Administration (FAA) regulations. Table 2 summarizes the specifications of the cameras and the obtained spatial resolution for the UAV surveys performed.

#### 2.2.3. Data Processing

The UAV imagery obtained for this research was processed using Pix4Dmapper Pro software (Pix4D SA, Prilly, Switzerland). The orthomosaics were generated by “stitching” all the overlapping images together obtained with a particular camera during a UAV survey. The end product was a set of orthomosaic image maps of the complete site survey area from the VIS-C, MS, and TIR cameras. While the VIS-C imagery was the bird’s-eye view of the survey area, as can be seen by the human eye in true color, the MS and TIR imagery was generated in grayscale with the lighter shaded areas representing either a greater reflection of green, red, red edge, or NIR EM radiation or a greater emission of TIR radiation, respectively. The orthomosaic images were post-processed using ArcMap 10.6 desktop software (ESRI, Redlands, CA, USA), where annotations were added and the images were then saved in a manageable format. Further processing was accomplished using the free public access GNU Image Manipulation Program (GIMP) 2.10.12 image editor. This software allowed for adjustment and enhancement of exposure, color levels, saturation, contrast, sharpness, image size, and resolution.

**Table 2:** Camera specifications and spatial resolution for the UAV flights performed at approximately 117 m height.

Camera	Sensor	Center Wavelength(s) nm	Bandwidth nm	Resolution cm/Pixel
S.O.D.A	RGB *	450, 520, and 660	~300	2.8
Sequoia	RGB *	470, 550, and 660	~300	3
	Green	550	40	11
	Red	660	40	11
	Red Edge	735	10	11
	Near-Infrared	790	40	11
thermoMAP	Thermal Infrared	10,000	3000	22

* RGB refers to red–green–blue comprising the visible wavelengths of the electromagnetic spectrum.

### 2.3. GPR Equipment, Survey, and Data Processing

#### 2.3.1. Equipment

The GPR used in this study was a time-domain SmartCart Noggin 250 MHz system (Sensors and Software Inc., Mississauga, ON, Canada; Figure 4b). These center-frequency antennas were earlier proven to be the most optimal for agricultural subsurface drainage mapping pertaining to the drainage pipe diameters and their installation depths typically encountered in the USA [14,15,16,26,29]. The antennas used were shielded and had a frequency bandwidth ranging between 125 and 375 MHz. An integrated odometer wheel on the SmartCart unit measured distance along the traverse to trigger the GPR data acquisition for uniform data coverage. The GPR equipment settings included parameters such as station interval, stacking, radar velocity, and depth of investigation, and were input using the digital video logger (DVL) at the beginning of the surveys. The station interval was set to 5 cm (i.e., the distance between the consecutive measurement points) and the data were stacked (averaged) using 32 signal traces at each measurement point to reduce the background noise and increase the signal PD. A time-domain reflectometer (Field Scout TDR-300; Spectrum Technologies, Inc., East Plainfield, IL, USA) with 20 cm waveguides was used to measure the soil water content at each field to determine the RDP for preliminary EM wave velocity estimation before performing the GPR surveys [48,68]. Based on the velocity estimates, the GPR two-way travel time for each signal trace was set to provide a depth of investigation of around 1.6–2.2 m at each site. Note that this is the depth of interest concerning drainage pipe location, as they are usually installed within the top 1.5 m of the subsurface. A Smart-V1 (NovAtel Inc., Calgary, AB, Canada) GNSS receiver was used to geo-reference the GPR data.

#### 2.3.2. Survey Information

At Sites-1, -2, and -4, the GPR data were collected simultaneously with the recent UAV flights along a few lines perpendicular to the expected drain line orientation and in a random fashion where the drainage pipes’ existence and orientation were unknown (see later in Section 3.2). As the DVL has a display screen that provided a real-time GPR cross-section view of the subsurface, spiral and serpentine transects were performed at locations where the drain line signature was ambiguous to ensure the hyperbolic patterns were actually caused by drain lines and to determine their orientation. This was conducted to help eliminate the false positives, i.e., isolated hyperbolic patterns caused by solitary buried objects such as rocks and cavities, akin to the approach proposed by Allred et al. [26]. At Site-3, since the expected drainage pattern was unknown, the preliminary survey was performed on 12 July 2018 (on the same day as the recent UAV flights) using a few transects along the edge of the site to determine if this field is potentially drained. The most recent GPR survey was performed on 10 December 2019, along a few parallel transects covering both the edge and the center of the field to map the drainage pipes’ location. Additionally, here, the spiral and serpentine transects were employed at a few locations to confirm the existence of the drain lines and to determine their orientation. Further, as a general protocol, at each site, the data were collected as a “lineset” constituting multiple lines to limit the file size and prevent the files from being corrupted.

#### 2.3.3. Data Processing

The GPR data were processed using EKKO Project V5 Suite software (Sensors and Software Inc., Mississauga, ON, Canada). The processing steps involved: (1) application of a signal saturation correction filter (i.e., Dewow) to remove slowly decaying low-frequency noise, (2) application of a background removal filter to remove the background noise, especially ground clutter which is a laterally continuous signal caused by cross-talk between antennas [48], and (3) utilization of a spreading and exponential calibrated compensation gain function to amplify potential GPR drainage pipe responses. The velocity was readjusted by using the reflection hyperbola curve fitting procedure in the EKKO Project software. This procedure was performed on multiple hyperbolic signatures at each site to refine the velocity estimate. Specific care was taken to include only the cases where the GPR transect was perpendicular to the drain line directional trend. The potential drainage pipe responses were marked on all GPR profiles using the LineView and Interpretation modules in the EKKO Project software. As a quality control measure, all the interpretations were later verified in MapView to check if a similar pipe-like response was recorded when performing random, spiral, and serpentine transects or surveys along parallel transects spaced few meters apart. Later, the interpretations were exported as a spreadsheet and KMZ files. The spreadsheet contained information on the latitude, longitude, and depth at which the drainage pipe responses were recorded. The KMZ file allowed the overlay of the GPR measurement transects and the possible pipe locations on the Google Earth aerial imagery.

Additionally, to further interpret the GPR data, average trace amplitude (ATA) plots were generated at each site using the EKKO Project software. The ATA plot represents the decay of the average signal amplitude with time. It is created by averaging all the GPR traces collected in each line of the lineset after rectification, i.e., by considering the absolute valued signal. As the receiver of the Noggin GPR system starts recording the data a few nanoseconds before the transmitter fires, it is possible to assess the background radio frequency noise floor and quantify the GPR PD with the help of ATA plots. Furthermore, they also provide important information on coherent system noise, flat-lying reflectors, clipped GPR signals, signal attenuation, and the choice of appropriate gain function [31,69,70].

## 3. Results and Discussion

### 3.1. UAV Results

**Figure 5:**
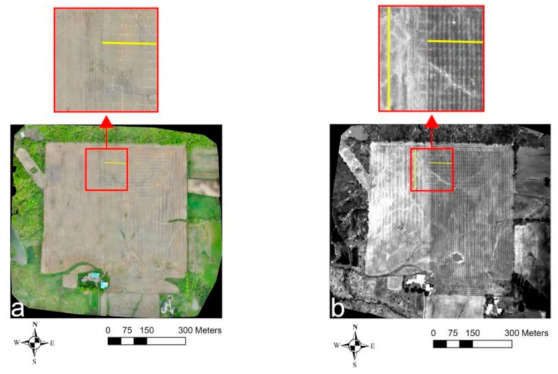

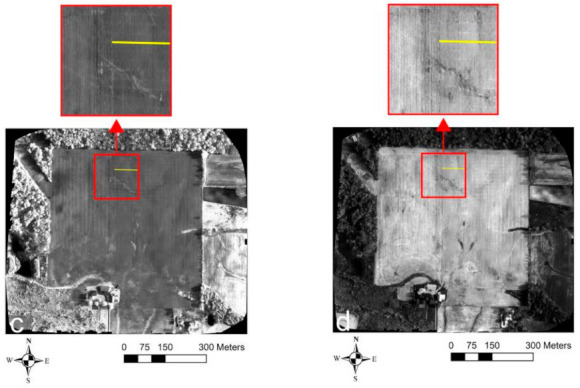
Site-1 UAV survey results from 6 May 2019: (**a**) VIS-C, (**b**) TIR, (**c**) NIR, and (**d**) red orthomosaic imagery. Solid yellow lines indicate the drain lines’ directional trend.

Figure 5 shows the VIS-C, TIR, NIR, and red orthomosaic imagery generated from the UAV flights carried out at Site-1 on 6 May 2019. Here, in all the orthomosaics, the drain lines showed up as lighter shaded linear features due to a higher reflectance in the VIS, NIR, and red bands and a higher emitted TIR radiation from the drier soil above the drain lines compared to the wetter soil in between the drain lines. In the VIS-C imagery (Figure 5a), to the eastern part of the field, the drainage pattern depicted by the lighter shaded linear features aligned well with the contractor’s drain map (Figure 3a) and the Google Earth imagery (Figure 2a). However, the drainage pipes’ signature fades out in the southern part of the field. In the north-central region depicted in the enlarged inset of the TIR imagery (Figure 5b), the drain lines trend in the east–west direction to the eastern part and north–south direction within the western part. Additionally, a few random drain-like signatures were observed trending in the northeast–southwest and northwest–southeast directions. While a similar response was observed in the VIS-C, NIR, and red enlarged insets (Figure 5a,c,d) for the east–west-trending drain lines in the eastern part, the north–south-trending drain lines to the western part became invisible.

Towards the western part of the entire field, as evident from the TIR orthomosaic (Figure 5b), the drain line features orient in the north–south direction and align with the known set of north–south-trending corrugated plastic tubing installations [39]. No regular drain-like features were observed in the southwestern part of the field in any of the orthomosaics. Although TIR imagery captured the drain lines to the western part of the field, noticeably, farm field operations (e.g., wheel tracks due to harvest, tillage, sowing equipment) also caused a similar lighter shaded linear feature response along the north–south direction in this field (Figure 5b). Therefore, the drainage pipes’ response trending in the north–south direction was overshadowed and hard to isolate when compared to the east–west-trending drain lines. In the NIR and red orthomosaic imagery (Figure 5c,d), similar to the VIS-C orthomosaic (Figure 5a), the response of the north–south-trending drainage pipes to the eastern part of the field, though visible to a lesser extent, was easier to discern as the signature caused by farm field operations was suppressed.

**Figure 6:**
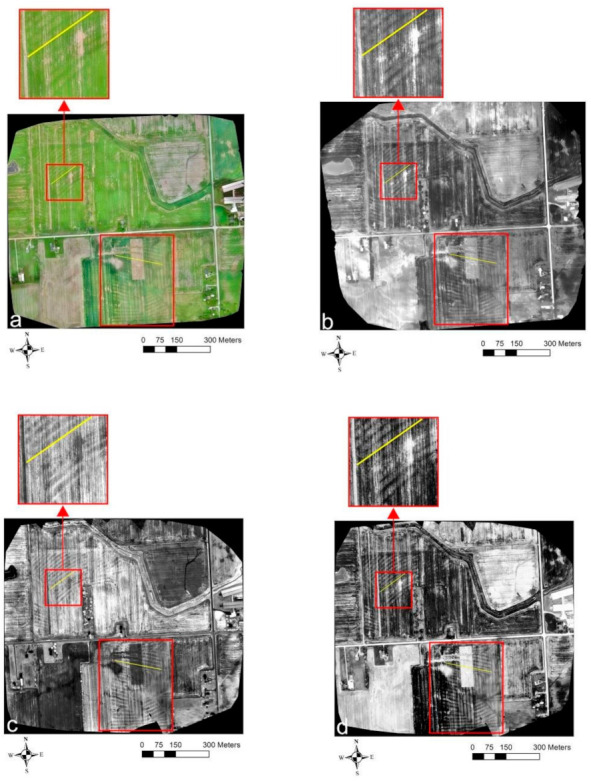
Site-2 UAV survey results from 21 May 2019: (**a**) VIS-C, (**b**) TIR, (**c**) NIR, and (**d**) red orthomosaic imagery. Solid yellow lines indicate the drain lines’ directional trend. The red boundaries mark the regions where the drainage pipe signatures were evidently visible.

**Figure 7:**
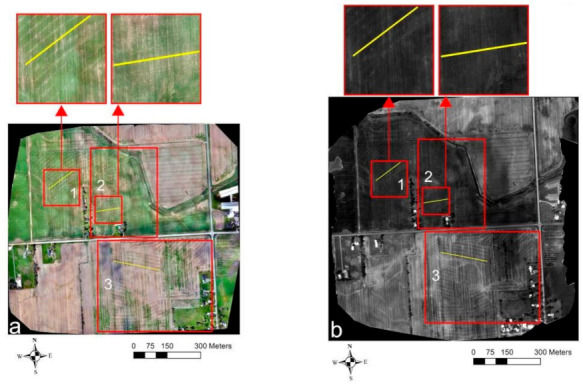
Site-2 UAV survey results from 7 May 2018: (**a**) VIS-C and (**b**) TIR orthomosaic imagery. Solid yellow lines indicate the drain lines’ directional trend. The red boundaries mark the different regions where the drainage pipe signatures were evidently visible.

Figure 6 and Figure 7 show the results obtained from the recent (21 May 2019) and older (7 May 2018) UAV surveys at Site-2. Figure 6 shows the drain lines’ location was revealed as the differences in crop establishment in the western part of the field north of the road and central part of the field south of the road due to the established cereal ryegrass cover crop during the recent surveys. In the VIS-C orthomosaic (Figure 6a), the crops directly on the top of the drain lines were comparatively greener and well developed as compared to the crops in between the drain lines. As discussed earlier, this is because the drainage systems modulate the water table, providing optimal soil–water–air conditions for plant growth directly above the drain lines [35,40,45]. Interestingly, the TIR (Figure 6b) and different bands of MS imagery (NIR and red—Figure 6c,d, respectively) also worked well even with the cover crop to discern the drain line locations. In the TIR orthomosaic (Figure 6b), the drain line features showed up as colder anomalies (i.e., darker shaded linear features) which is coherent with the observations noted by earlier studies that TIR imagery can be useful to indicate the spatial variation in crop health/stress [62,63,64]. While the drain lines show up as lighter shaded linear features in the NIR orthomosaic, the opposite was true in the red band orthomosaic of the MS imagery. This is because the stressed vegetation has a different spectral reflectance signature (i.e., higher reflectance in the red and lower reflectance in the NIR region of the EM spectrum) as compared to the healthy vegetation [71]. A more or less uniform drain line signature was observed across the entire field in all the orthmosaics generated from the recent surveys.

The VIS-C and TIR orthomosaics generated from the older surveys (Figure 7) when the soybean/corn crop (see Table 1) was in the early stage of development can broadly be classified into three regions based on the observed drainage pipe signatures. In Region-1, shown as the enlarged inset to the western part of the field north of the road where the soybean crop was planted, bare ground was exposed with less to no crop development. This revealed the drainage pipe locations in the VIS-C orthomosaic (Figure 7a), which could happen when the drain lines dewater the field quickly, providing less optimal conditions for crop development. In the TIR orthomosaic (Figure 7b), the same drain lines showed up as hotter anomalies (i.e., lighter shaded linear features). Contrarily, in Region-2 located towards the center of the field north of the road, the drainage pipes showed up as greener linear features in the VIS-C orthomosaic (Figure 7a) with early soybean crop establishment, as was the case in the recent survey of the VIS-C orthomosaic ( 6a). Here, the same drain lines showed up as colder anomalies in the TIR orthomosaic (Figure 7b), which was again similar to the drainage pipe signature observed in the recent survey of the TIR orthomosaic (Figure 6b). An enlarged inset is also provided from a small part of Region-2 to showcase the drainage pipe signature (Figure 7). Interestingly, although a similar drainage pipe response was observed in Region-2, as in the recent surveys, no drainage pipes were identified in the recent surveys where Region-2 was located. In Region-3, located towards the south of the road where corn was planted and the bare ground was exposed, the drainage pipes showed up as linear features with a higher reflectance in the VIS-C orthomosaic due to differences in spectral reflectance between dry and wet soils [56,57]. Here, the drainage pipes showed up as hotter anomalies in the TIR orthomosaic due to the difference in thermal inertia and emissivity between the dry and wet soils [59,60,61].

**Figure 8:**
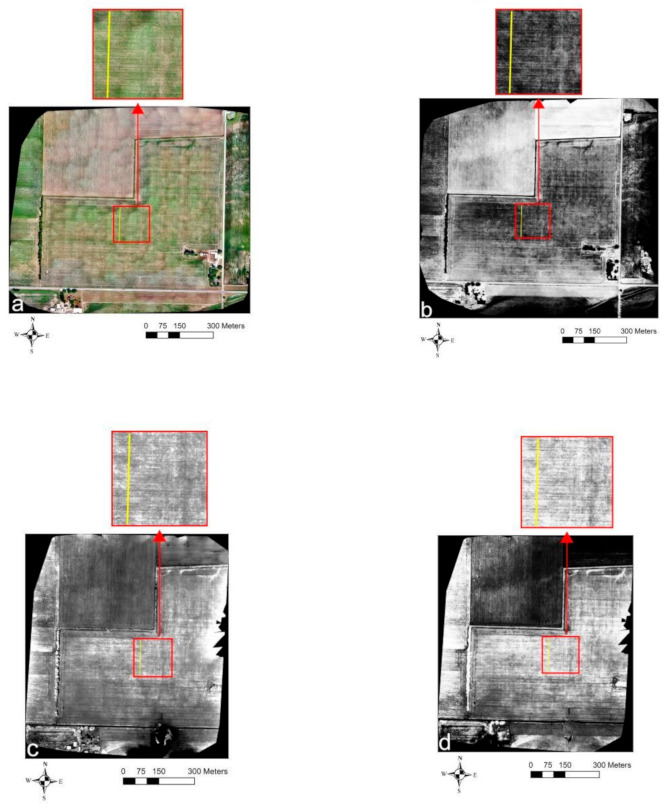
Site-3 UAV survey results from 12 July 2018: (**a**) VIS-C, (**b**) TIR, (**c**) NIR, and (**d**) NDVI orthomosaic imagery. Solid yellow lines indicate the drain lines’ directional trend.

**Figure 9:**
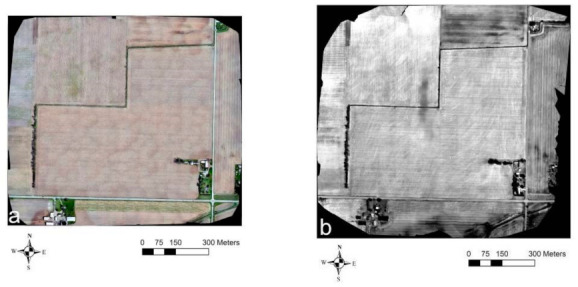
Site-3 UAV survey results from 7 May 2018: (**a**) VIS-C and (**b**) TIR orthomosaic imagery.

At Site-3 (Figure 8), the more recent UAV flights carried out on 12 July 2018 showed a subtle signature of drainage pipes as differences in the development of the soybean crop. Again, as was the case in recent UAV VIS-C surveys at the Clayton site (Figure 6a), the crops directly overlying the drain lines were comparatively greener and well established as compared to the crops in between the drain lines (Figure 8a). Synonymously, an alike response was also observed in the TIR and NIR orthomosaics with the drain lines, respectively, showing up as darker and lighter shaded linear features (compare Figure 6b,c with Figure 8b,c). Moreover, the drain lines also showed up as lighter shaded linear features in NDVI (Figure 8d), which again proves the supposition that crops overlying the drain lines are often in better health due to optimal soil–water–air conditions. Figure 9 shows the VIS-C and TIR orthomosaics from the older surveys carried out on 7 May 2018, on bare ground conditions. Here, as was also the case in the historically recorded Google Earth imagery, none of the drain-like patterns were identified in both the images. This could lead to a misinterpretation that the field is potentially undrained with subsurface drainage systems.

**Figure 10:**
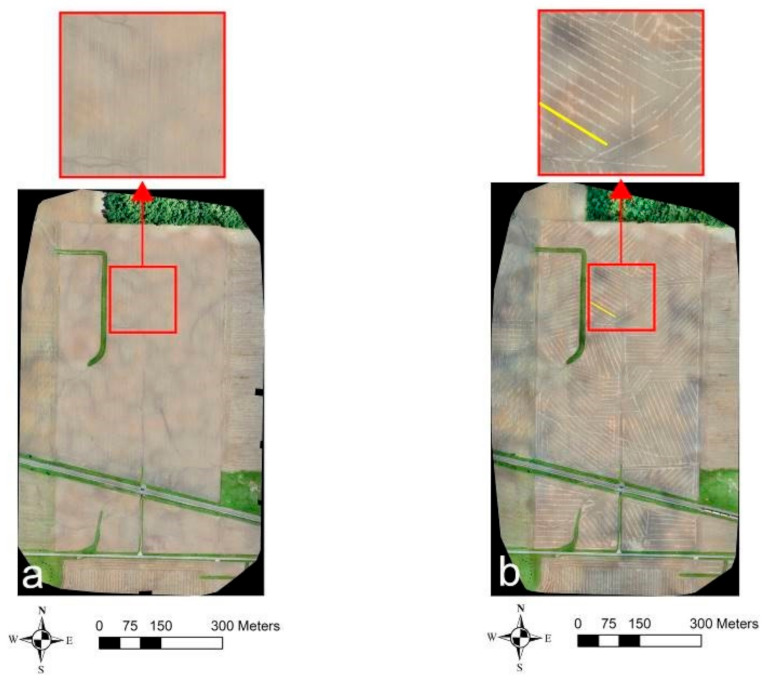

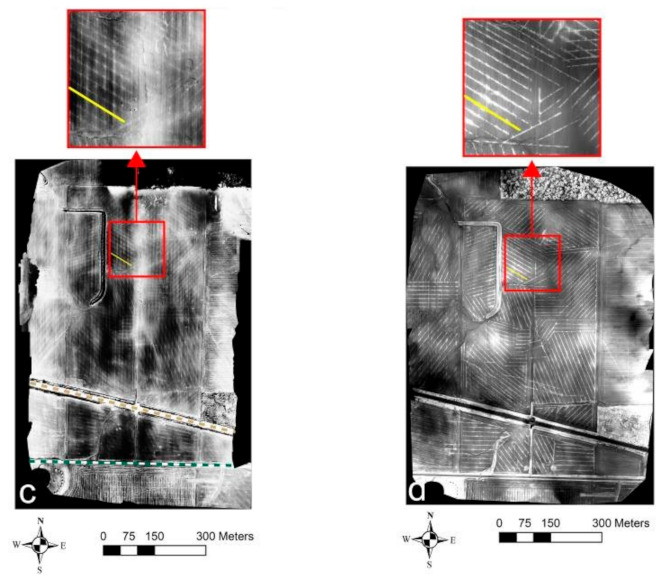
Site-4 UAV survey results from 21 June 2019: (**a**) VIS-C 1, (**b**) VIS-C 2, (**c**) TIR, and (**d**) red edge orthomosaic imagery. Solid yellow lines indicate the drain lines’ directional trend. The first set of UAV surveys with the VIS-C and MS camera payloads was performed approximately at 10:30 a.m., while the second set was performed seven hours later. The UAV TIR survey was performed between 11:00 and 11:30 a.m. Railway crossing and a county road are highlighted, respectively, by brown and green dashed lines in 10c. Part of the results (10a and b) was presented in [40].

At Site-4, the VIS-C imagery captured at 10:30 in the morning on extensive bare ground conditions after a large rainfall event (4.2 cm) the previous night did not reveal any indications of the drainage pipe signature (Figure 10a). However, the VIS-C imagery captured approximately seven hours later revealed most of the drainage pipe locations (Figure 10b). As also noted by Allred et al. [40], this implies that the water overlying the drainage pipes had sufficiently drained in just seven hours from morning to afternoon for the drainage response to show up in the VIS-C imagery (Figure 10b). This was also true for the flights performed with the MS camera approximately seven hours apart at this site, as the drain lines’ response showed up well in the second flight when compared to the first (Figure 10d). Interestingly, even under the undrained conditions, the TIR survey performed at approximately 11:00 in the morning was able to capture most of the drainage pipe locations as the drain lines showed up as lighter shaded linear features (Figure 10c). However, here, the farm field operations trending in a north–south direction to the north of the railway crossing (brown dashed line in Figure 10c) and a more east–west direction between the railway crossing and the county road (green dashed line in Figure 10c) also caused lighter shaded linear features, making it hard to distinguish the drainage pipes’ response.

### 3.2. GPR Results 

**Figure 11:**
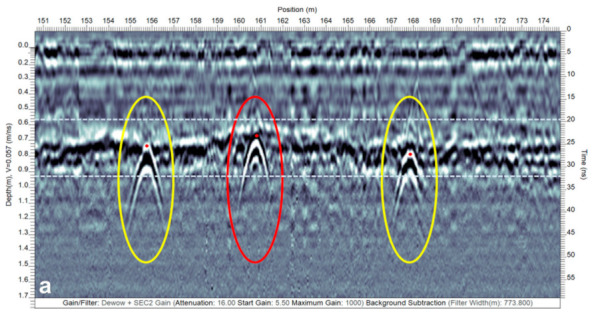

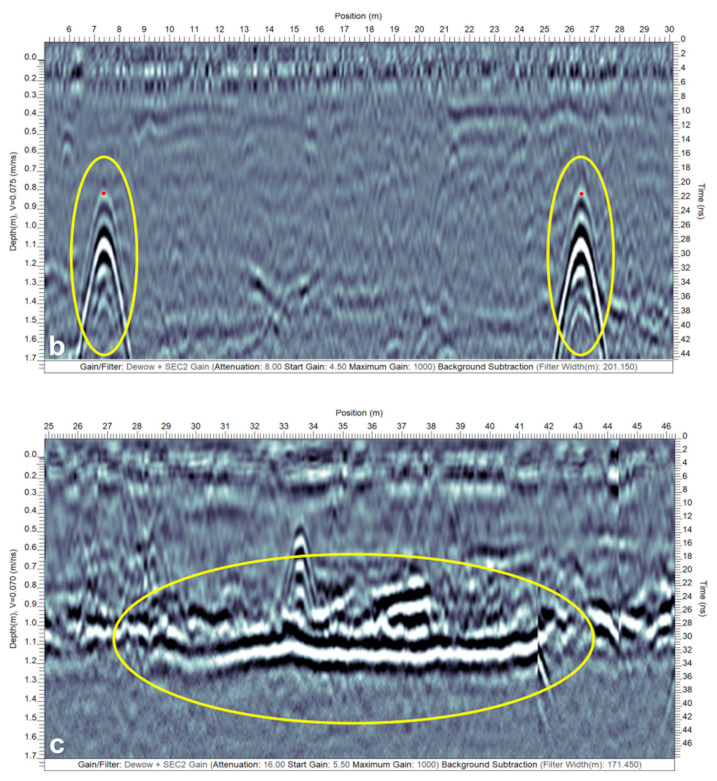
Drainage pipe response on the GPR profiles: (**a**,**b**) single (yellow and red) and dual (yellow) reflection hyperbolas result when the GPR traverse is perpendicular to the drainage pipe orientation (from Sites-1 and -3, respectively), and (**c**) banded linear feature (yellow) results when the GPR traverse is over the top and along the trend of the drainage pipe (from Site-2). In (**a**), the strong reflections from the water table due to a high RDP contrast are highlighted within the light blue dashed lines. Note that the red dots correspond to the actual location or top of the drain lines.

The GPR wave velocity was estimated to be 0.057 m ns^−1^ at Site-1, 0.070 m ns^−1^ at Site-2, 0.080 and 0.075 m ns^−1^, respectively, in the preliminary (12 July 2018) and recent (10 December 2019) surveys at Site-3, and 0.052 m ns^−1^ at Site-4 using the reflection hyperbola curve fitting procedure in the EKKO Project software. Based on the velocity estimates, the soil at Site-3 during the GPR surveys in both instances was drier than the soil at Sites-1, -2, and -4. Figure 11shows three GPR profiles from different sites to explain a few interesting drainage pipe signatures. Figure 11a,b show single and dual hyperbolic responses from Sites-1 and -3. As stated earlier, a hyperbolic response is caused by a point size object, i.e., either when the GPR traverse is perpendicular or at a somewhat modest to large angle (i.e., 15° < x° < 90°) to the drain line orientation. The response could also be due to any other point size object such as rocks and cavities. This is due to the fact that the GPR signal propagates into the subsurface as an elongated cone of energy and “sees” buried features both in front of it and behind it. Thus, the arrival time of reflections retraces a hyperbolic pattern with the apex of the hyperbola coinciding with the location and depth of the object.

In relation to the drainage pipes, the hyperbolas are horizontally compressed when the angle is closer to 90° and become horizontally spread out as the angle deviates from 90° and is closer to 15° [26]. Moreover, a single hyperbolic response is observed when the drainage pipe is filled with air or only partially filled with water (Figure 11a), whereas a dual hyperbolic response results from an undrained drainage pipe completely filled with water as the GPR wave travels nine times slower (Figure 11b) [16,50]. In case the latter response is observed, it is possible to estimate the drainage pipe diameter, and the bottom hyperbolic response is usually stronger than the top (see Figure 11b). This is because less energy is reflected from the soil–water interface when the GPR wave interferes with the top of the drainage pipe compared to the water–soil interface when the wave exits from the bottom of the drainage pipe [16]. Technically speaking, the response from dual to single hyperbolas is gradational as the drainage pipe transitions from being completely water-filled to air-filled. Yet, this gradation is often impossible to track for the typical drainage pipe diameters (i.e., 50–100 mm for the laterals) both due to weaker reflections from the top of the pipe and constructive interference between the reflections from the top and bottom of the pipe given an insufficient time gap. This reasoning causes partially water-filled drainage pipes to often generate a single hyperbolic response.

Nevertheless, it is still possible to differentiate between the drainage pipes filled with air and those partially filled with water as a polarity flip is observed in the single hyperbolic responses caused by them. In Figure 11a, the hyperbolic response surrounded by a red ellipse is typically expected in case the drainage pipe is filled with air. Contrarily, a reverse polarity (yellow ellipses) is expected when the pipe is partially filled with water. Here, the water table also caused strongly pronounced reflections, as depicted within the interval highlighted by the light blue lines. Furthermore, care should be administered while interpreting the response caused by partially water-filled drainage pipes as a similar response is expected (i.e., the polarity of the EM wave is preserved) in case the pipes are metallic such as the underground utility installations (e.g., power lines). However, since the RDP of metals is infinite, the GPR wave ceases to propagate and results in noise beyond the pipe depth in the GPR vertical profile, thereby providing a means to differentiate them.

Single hyperbolic responses were mostly observed at Sites-1, -2, -3 (preliminary survey), and -4, while the dual hyperbolic responses were observed only at a few locations in the recent surveys at Site-3. Hence, this shows even though Site-3 was drier in both instances when compared to Sites-1, -2, and -4, there were a few locations in the recent surveys at Site-3 where the drain lines were undrained and completely filled with water. In the scenario when the angle is smaller (i.e., x° < 15°) between the GPR traverse and the drain line orientation (i.e., almost parallel), and the GPR traverse happens to occur right above the drainage pipe location, the response of the drainage pipe shows up as a banded linear feature with the top of this feature corresponding to the top of the drainage pipe [26]. Figure 11c shows an example of a banded linear feature from Site-2 due to a GPR traverse that was over the top and along the trend of the drainage pipe. At all the sites, the hyperbolic responses were typically observed at 0.5–1.1 m depth and the drainage installation depths varied between different sites.

**Figure 12:**
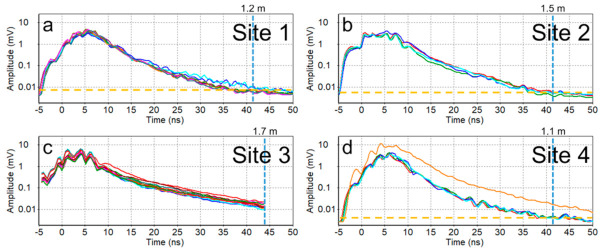
Average trace amplitude plots marked (in blue dashed lines) with approximate GPR signal penetration depths (PDs): (**a**) Site-1, (**b**) Site-2, (**c**) Site-3 (from the recent survey conducted on 10 December 2019), and (**d**) Site-4. Yellow dashed lines mark the approximate threshold voltage representing the radio frequency noise floor. Note that the PDs expressed in the distance (m) are marked on the top of each sub-figure.

To further explore the GPR PD at different sites, Figure 12 shows the ATA plots derived from the acquired GPR data. Except for Site-3, at the rest of the sites, the ambient radio frequency signal (commonly regarded as the “noise floor”) during the time of the surveys is depicted as yellow dashed lines. The two-way travel time (or depth) at which the GPR signal reaches this noise floor is considered as the PD, which is converted to depth expressed in distance based on the velocity estimates. The GPR PD was shallower (1.1 m) at Site-4 (Figure 12d) in comparison to Sites-1, -2, and -3 (Figure 12a–c). At Site-3, as the time window over which the GPR data were collected was narrower in the old and recent surveys, it was not possible to quantify the GPR PD. However, it can be reasonably assumed to be greater than 1.5 m, i.e., the maximum depth for typical drainage installations. As expected, the GPR PDs (Site-3 > Site-2 > Site-1 > Site-4) varied in the same order as the observed GPR wave velocities (Site-3 > Site-2 > Site-1 > Site-4) at the four sites. This can be attributed to an increase in the soil EC (in turn, the GPR signal attenuation) with the water content (i.e., inversely proportional to the GPR wave velocity) as the soil texture and water content are the predominant factors that control the soil EC in non-saline soils [72,73]. Hence, based on the ATA plots alone, we can expect greater success in drain line mapping at Site-3 (Figure 12c) and the least success at Site-4 (Figure 12d). Moreover, as can be seen in Figure 12d, the orange average trace of a line of the lineset at Site-4 outlies the rest of the average traces. This was a line collected across the railway crossing (highlighted by the brown dashed line in Figure 10c). Hence, the data were noisy due to interference with the railway installations (see Supplementary Material Figure S1). This further illustrates the importance of ATA plots for a comprehensive understanding of the collected GPR data.

**Figure 13:**
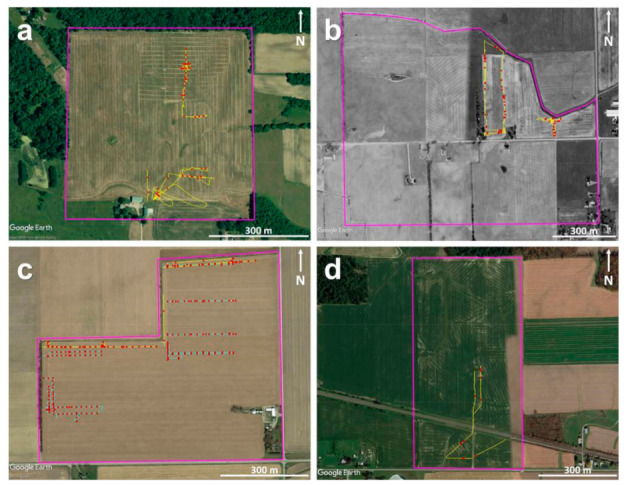
Potential drainage pipe locations marked (with red dots) on the GPR survey transects (yellow and cyan) overlaid on the Google Earth aerial images (same as in Figure 2): (**a**) Site-1, (**b**) Site-2, (**c**) Site-3, and (**d**) Site-4.

Figure 13shows the potential drainage pipe locations (marked with red dots) on the GPR transects where hyperbolic signatures were detected at the four sites. A total of 81 locations were identified at Site-1 (Figure 13a), 104 locations at Site-2 (Figure 13b), 90 and 194 locations, respectively, in the preliminary and recent surveys at Site-3 (Figure 13c), and only 12 locations at Site-4 (Figure 13d). This was consistent with assumptions made earlier based on the ATA plots, more specifically, the observed PDs at the different sites. Although there were a few locations where the GPR traverse was exactly over the top and along the trend of the drain line by chance, these locations where corresponding banded linear features were observed in the GPR profiles were not marked in the figure. This is because it is fairly uncommon without any prior knowledge of drain line locations to traverse exactly on top and along the trend of a drain line while surveying large farm field areas. 

Figure 13a shows that most of the potential drainage pipe locations marked on the GPR transects at Site-1 overlie exactly on the top of the drain line features visible in the Google Earth imagery. A slight offset was observed at a few locations and can be associated with the positional accuracy of the Google Earth imagery or the GNSS receiver used accompanying the GPR surveys. The marked points followed a line in the spiral, serpentine, and randomly fashioned transects used when the drainage pipe signature was ambiguous in the real-time GPR cross-section view on the DVL. The use of these transects clearly identified that the signature was actually caused due to a drainage pipe and, at the same time, assisted to determine its orientation. A few solitary locations were also identified as potential drainage pipe locations in the random transects to the southern part of the field. However, it was difficult to demarcate if this signature was caused by drainage pipes (or remnants) or other solitary objects in the soil. Moreover, GPR was also successful in finding the locations of drainage pipes to the southeastern part of the field that were visible in the Google Earth imagery (Figure 13a) and the contractor’s plan (Figure 3a) but were not identified in the UAV imagery (Figure 5).

At Site-2 (Figure 13b), drainage pipe signatures were not visible on the Google Earth imagery where the GPR transects were measured. Therefore, it was difficult to ascertain if the marked locations represent actual drainage pipes. Nevertheless, similar to Site-1, there were locations where the marked points form a line on the spiral, serpentine, and randomly fashioned transects, showcasing the potential locations and the trend of the drainage pipes. Again, the locations where a solitary response was observed can either be due to a drainage pipe or any unknown object. However, based on the GPR data alone, it can be reasonably assumed that also this part of the field is subsurface drained.

At Site-3 (Figure 13c), the preliminary surveys conducted along the edge of the field revealed that this field might as well be subsurface drained. The recent surveys at this site focusing on measuring along parallel transects as well as using spiral and serpentine transects indeed confirmed this supposition, as many pipe-like responses were recorded by GPR. Similar to spiral and serpentine transects, parallel transects spaced few meters apart were also helpful to ascertain if the response was caused due to drainage pipes and to determine their orientation. Moreover, parallel transects spaced a few meters apart also allowed the determination of the drainage network pattern and orientation without the necessity of performing a dense GPR transect grid over the field. This was similar to the approach proposed by Allred et al. [26] while surveying large farm field areas. As can be seen in Figure 13c, the drain lines at Site-3 trend in a north–south orientation, and the drainage network pattern might as well be parallel (Figure 1a).

**Figure 14:**
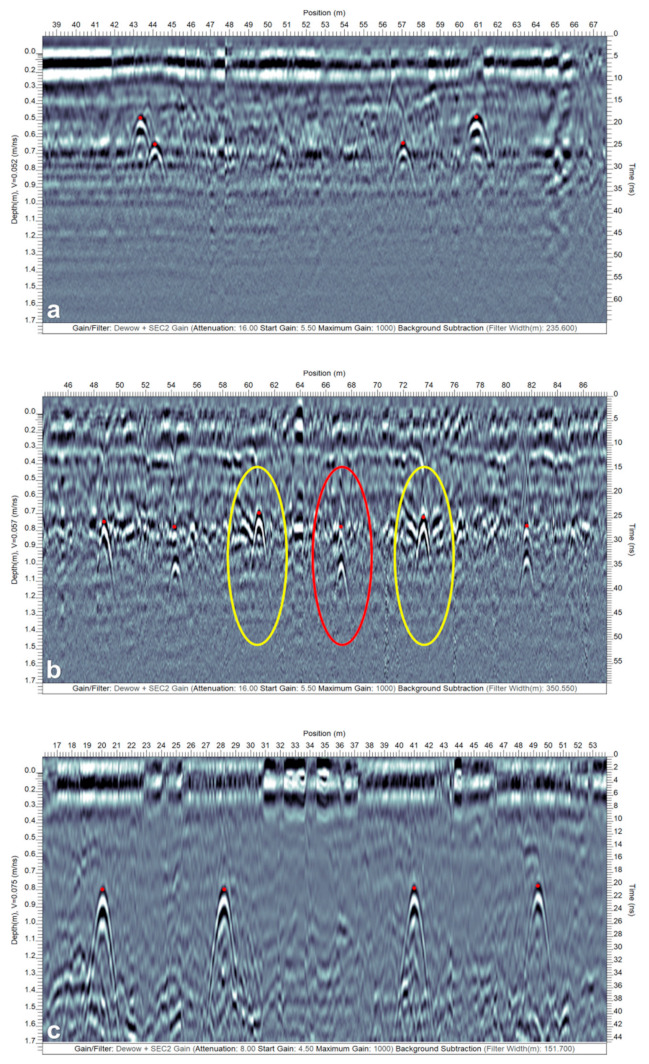
Drainage pipe response on the GPR vertical profile depicting closely spaced hyperbolic features from two different depths at: (**a**) Site-4 (from Region-2 of Figure 15d), potentially caused due to drainage pipes from different generations of installations, (**b**) Site-1 (from Region-1 of Figure 15a), caused due to the same drainage pipe probably with undrained water, but the first hyperbolic response being very weak in some instances obscures its visibility. Note that the latter profile can potentially be misinterpreted as a response caused due to two co-existing drainage pipes at different depths. (**c**) depicts the drainage pipe response from Site-3 (from Region-2 of Figure 15c). Here, the closely spaced hyperbolic features at a similar depth are caused due to the spiral and serpentine transects employed.

At Site-4 (Figure 13d), the few potential drainage pipe locations identified and marked on the GPR transects closely matched with the drainage pipe features visible on the Google Earth imagery. However, at some places, the marked locations were very close spatially and the hyperbolic responses were identified at different depths (Figure 14a), which can be due to co-existing drainage pipes overlying on one another that date back to two different installation periods.

### 3.3. Complementary Nature of the UAV Imagery and GPR and Combined Interpretation

**Figure 15:**
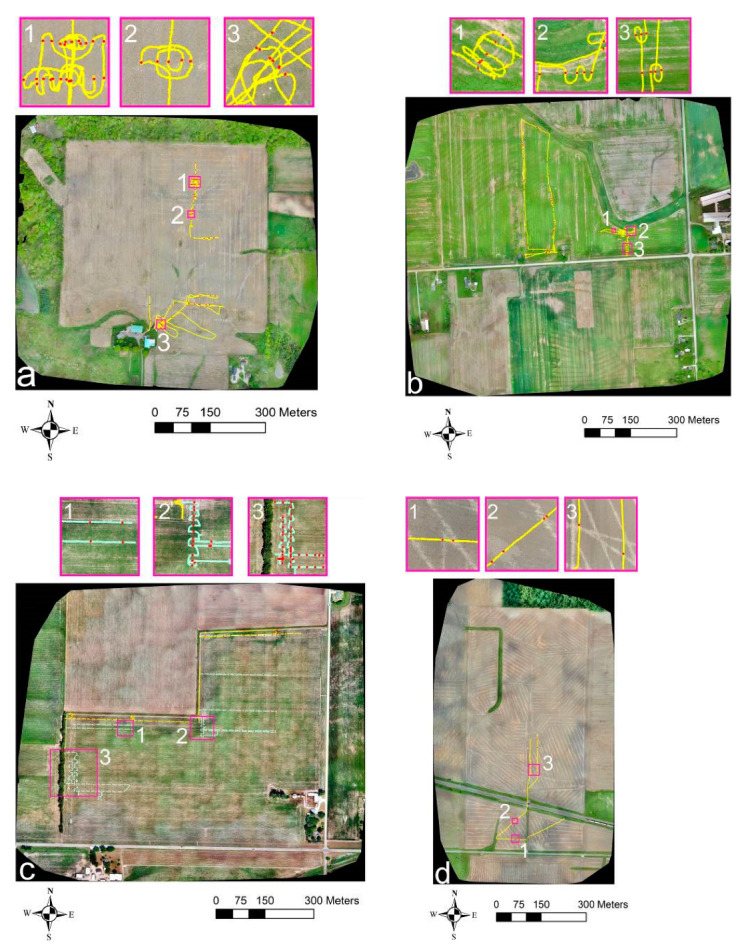
Potential drainage pipe locations marked (with red dots) on the GPR survey transects (yellow and cyan) overlaid on UAV VIS-C orthomosaic imagery: (**a**) Site-1, (**b**) Site-2, (**c**) Site-3, and (**d**) Site-4. The pink boundaries mark the regions of interest.

A combined interpretation was made to understand the complementary nature of the UAV imagery and GPR for subsurface drainage mapping by overlaying the GPR transects and the potential drainage pipe locations marked on the UAV VIS-C imagery acquired at the four sites (Figure 15). The VIS-C orthomosaic imagery was used in this example, as it is more generally applicable and readily available information in comparison to the MS and TIR imagery. Figure 15a shows the UAV imagery and GPR data from Site-1 with a few interesting regions marked. Regions-1 and -2 show examples of the spiral and random transects used to confirm if the linear features were related to buried drainage pipes as well as reaffirming their orientation. The GPR profile (Figure 14b) from Region-1 at Site-1 (Figure 15a) looked as if the signature was actually caused by two drainage pipes located at two different depths (i.e., at 0.75 and 1.0 m), which, if visible in the UAV imagery, might only produce a single linear feature. Again, this can happen when the drainage pipe systems from different generations are spatially overlaid on the top of one another or not too far apart. This might mislead to an indefinite conclusion based on the UAV imagery alone. However, here, a closer look into the GPR profile (Figure 14b) revealed that the signature was actually caused due to the same drainage pipe probably with undrained water; i.e., a dual hyperbolic response was observed at some locations.On the contrary to Region-1 at Site-1, a drainage pipe response at similar depths was observed on the GPR profile from Region-2 (Figure 15a). Here, as well, the hyperbolic responses were spatially not too far apart (similar to Figure 14c) and were generated due to crossing the same drainage pipe multiple times in a spiral fashion. Note that this should not be mistaken as a response caused due to several closely spaced drain lines. Region-3 at Site-1 (Figure 15a) was located to the southern part of the field where the drain line signature, though visible in the Google Earth imagery (Figure 13a) as well as the contractor’s plan (Figure 3a), was faded off in the UAV imagery (Figure 5). Hence, at Site-1, based on the UAV imagery alone, only some of the known existing drainage pipes were mapped. Here, the GPR survey assisted both as a mapping and validation technique, providing the depth information of the drainage pipes.

At Site-2 (Figure 15b), the GPR transects were overlaid on the recent UAV imagery and, again, a few interesting regions were pointed out. Regions-1, -2, and -3 were located to the eastern part of the field north of the road where the drainage pipes were not visible either in the historical Google Earth imagery (Figure 13b) or in both the UAV surveys (Figure 6 and Figure 7) carried out at this site. Here, the use of spiral, serpentine, and random transects was helpful to delineate the drain line locations and to discern their orientation. Hence, at Site-2, GPR proved suitable as a mapping technique to the central and eastern parts of the field where the recently captured UAV imagery failed and additionally provided the depth information of the drainage pipes.

At Site-3 (Figure 15c), prior to the recent UAV flights with an established soybean crop, none of the UAV imagery captured on bare soil (Figure 9) or the historical Google Earth imagery (Figure 13c) was able to detect the drainage pipe signature or provide any hints that the field is potentially drained. Only the more recent UAV flights performed on an established soybean crop showed subtle signatures of drainage pipes as differences in crop development (Figure 8). Again, a few regions of interest were pointed out. Here, the use of parallel (Region-1), spiral, and serpentine (Regions-2 and -3) transects was helpful to validate that the field is drained and to determine the location and orientation of the drainage pipes. As an example, Figure 14c shows a GPR profile from Region-2 where closely spaced hyperbolic patterns were observed due to the serpentine transects employed. Here, as well, the hyperbolic responses were caused due to crossing the same drainage pipe multiple times. Moreover, the use of parallel transects also provided a general idea of the drainage network pattern and orientation at this specific site. Hence, at Site-3, based on the UAV imagery on bare soil (Figure 9) only, a possible misinterpretation could lead to the conclusion that this field is potentially undrained. Employing GPR in combination was helpful to both locate and validate the presence of the drainage pipes and retrieve their depth information, and, additionally, here, it provided an estimation of the drainage network pattern.

At Site-4 (Figure 15d), though the timing was crucial, the UAV imagery worked very well in discerning the drainage pipe locations, while GPR failed due to its limited PD. Here, performing a GPR survey on moderately dry soil might provide better results. Again, a few regions of interest were marked on the figure. While the locations marked on the GPR transects exactly overlie the drain lines’ signature visible on the UAV imagery at Regions-1 and -3, interestingly, multiple closely spaced hyperbolic responses at two different depths (see Figure 14a) were observed from Region-2 (Figure 15d). As discussed earlier, this might be because of spatially co-existing drain lines from different generations of installation. Hence, at Site-4, the UAV imagery was mostly successful in finding the drain lines and only a few drainage pipe responses were recorded by GPR.

Therefore, the site survey examples presented here depict both the extreme scenarios, comparing and contrasting the suitability of the two techniques. Overall, the UAV imagery proved to be a feasible and cost-effective technique, and GPR, when used appropriately in combination, proved to be a suitable complementary technique at three among four sites. When successful, GPR was useful as both a mapping and validation technique for drainage pipe detection and provided valuable information on their depth and orientation.

## 4. Recommendations and Future Work

At the four study sites visited, we were mainly successful at two sites and were only partially able to locate the drain line patterns at the other two sites using the UAV imagery. Except for one site (Site-3), the drainage pipe signature was also located in the historical Google Earth imagery at the other sites. Therefore, viewing all the historical imagery acquired to date could be a good starting point when planning to visit a new site. Moreover, as stated earlier, previous research recommends a rainfall event equal to or greater than 2.5cm [34] or 5.0 cm [33] and bare ground conditions for carrying out the aerial imaging surveys for subsurface drainage pipe mapping purposes. Our results suggest that adherence to the above thresholds is not strictly necessary as the drain lines’ signature shows up even when the soil is relatively dry, e.g., with a three days’ prior rainfall of 0.15 cm during the old surveys at Site-2 (see Table 1; Figure 7).

Given bare soil conditions, the drain lines’ signature shows up in the VIS-C and MS imagery because of the variation in spectral reflectance between wet and dry soil, while this relates to the differences in thermal inertia and emissivity in the TIR imagery. Contrarily, given the field is covered with a crop, the drain lines’ signature shows up as early crop establishment in the initial stages and due to differences in crop health or stress at the later stages. In particular, this could be important in soils as in Site-3 as no success was observed in locating the drainage pipes in the historical Google Earth imagery or the UAV imagery captured on bare ground conditions. Hence, concerning the prior rainfall and site surface conditions, the timing can be quite flexible for carrying out the UAV surveys depending on the soil type. However, when permissible, the UAV imagery surveys should be performed within two to five days after a significant (2.5 cm and greater) rainfall event on bare ground conditions (outside the growing season) or preferably with an early crop establishment to derive maximum success concerning drainage mapping purposes. Within the growing season, the timing could be flexible based on convenience and without an overriding consideration of the field wetness from prior rainfall. It is worth a mention that high relative humidity (RH > 60%) can, at times, adversely impact the TIR image quality due to atmospheric absorption and re-emission of the TIR radiation and hamper the generation of their orthomosaics [59,74,75].

In relation to the use of GPR, performing a few survey transects across a limited spatial extent proved useful for both mapping and validation purposes at three study sites. At the same time, the use of GPR can give valuable information on the depth of the drainage pipes. Hence, we recommend carrying out a few GPR transects in the direction perpendicular to the expected drain line orientation given this information is known in advance either from the historical Google Earth imagery or from the pre-existing drain maps acquired from the farmers/landowners/site managers. In this way, the GPR data can be acquired simultaneously along with the UAV flights. At the field sites where this a priori knowledge is unavailable, it is advisable to first carry out the UAV flights and do a rapid/low-resolution processing of the data using the Pix4Dmapper Pro software in order to check if any hints of the drain lines’ signature and trends show up in the UAV imagery. The GPR transects should then be performed accordingly. Moreover, performing rapid/low-resolution processing is always recommended as it acts as a field check quality control measure to ascertain if the UAV imagery collected is of good quality. This should also ensure a sufficient overlap during the flight plan to guarantee proper stitching later on while generating high-quality orthomosaics using full processing [76].

In case no traces of the drainage pipes are observed in the UAV imagery (stitched together with rapid/low-resolution processing), we advise carrying out a few parallel GPR transects spaced few meters apart or in mutually orthogonal directions to get a sense of whether the field is subsurface drained. Moreover, this would facilitate the generation of ATA plots to determine the GPR PD and comprehend if GPR would work for drainage pipe detection at a given field site [26,31]. This is because, although GPR worked well at Sites-1, -2, and -3, it should be kept in mind that conditions such as Site-4 do exist where EM waves attenuate relatively quickly when the soil has a high EC and the PD of the GPR signal could be smaller, thereby making it an unsuitable technique for drain line mapping [31]. In this relation, any a priori knowledge on the soil EC derived either from the existing soil maps or with the complimentary use of an electromagnetic induction sensor that is typically used for precision agriculture purposes [77,78] can also be useful to perceive whether GPR would work at a given field site. An EC less than 20 mS m^−1^ is preferred, permitting the expected GPR signal PD to be greater than 2 m in the best-case scenario, i.e., by considering that the decrease in the GPR signal strength is only due to ohmic signal attenuation [51,79].

Otherwise, some handheld soil moisture probes (such as TDR or a frequency-domain reflectometer) used to estimate RDP, e.g., CS655, SoilVUE10 (Campbell Scientific Inc., Logan, UT, USA), also facilitate the simultaneous measurement of the temperature and soil EC. Hence, the use of these handheld probes is recommended just before or at least concurrently with the GPR surveys at a few random locations spread over the entire field area for the preliminary EM wave velocity estimation and to discern the soil EC. Additionally, it can be difficult to traverse with GPR on a field covered with crop cover and achieve good antenna to ground coupling. Therefore, a general recommendation about the optimal timing concerning the prior rainfall and site surface conditions could be to conduct the GPR surveys two to three days after a rainfall event on moderately wet soil and bare ground conditions. This is because moist soils with air-filled drainage pipes provide a suitable RDP contrast and pose the best-case scenario for the drainage pipes’ detectability [14,55]. Nevertheless, the GPR surveys performed anytime thereafter on dry soil conditions are also expected to produce reasonable results.

**Figure 16:**
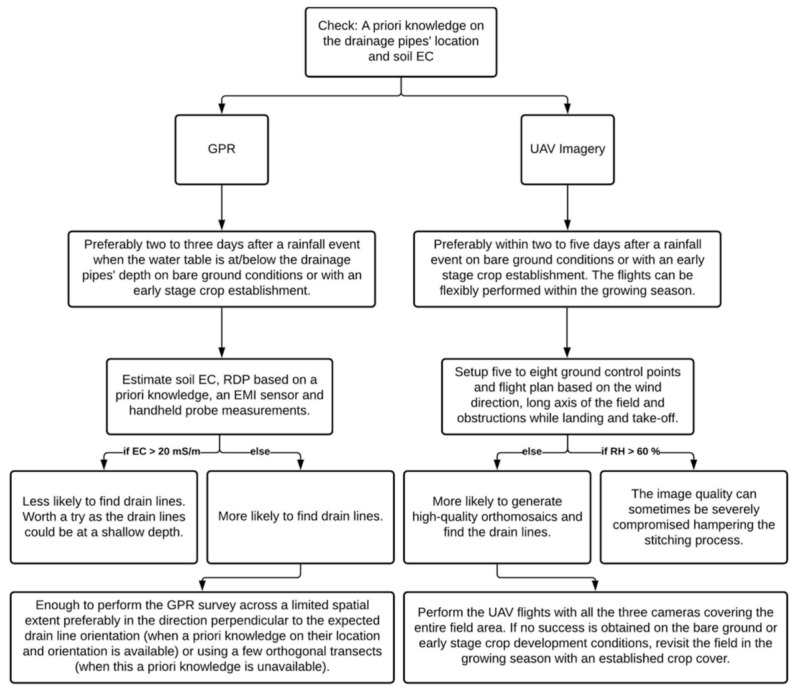
The decision support flowchart diagram providing guidelines for planning the subsurface drainage mapping surveys with GPR and UAV imagery.

When required to be operated in combination, visiting a site within two to five days after a significant rainfall on bare soil conditions is more likely an optimal timing for performing the drainage mapping surveys. However, it should be noted that soils such as Site-3 do exist where it is optimal to perform the UAV imagery and GPR surveys on surface conditions with the early establishment of crop cover for easier mobility of GPR. To summarize the above recommendations, guidelines are presented as a flow chart diagram in Figure 16 to support the decision-making process of farmers/site managers/land improvement contractors on the optimal survey timing for drainage mapping purposes. While following this general protocol is expected to deliver a maximum success rate, based on the authors’ experience, it should be noted that some excellent drainage mapping results were encountered in less than ideal conditions, and sometimes poor results were observed on more optimal conditions depending on the soil and crop type, drainage pipe depth and diameter, and other subordinate influential parameters.

Therefore, rigorous theoretical and practical studies are certainly warranted to fully comprehend the complex subsurface processes that affect the drainage pipes’ detectability. Hence, in efforts moving forward, future research should focus on understanding the dependence of these non-destructive techniques on soil type, crop residue, tillage practice, ground wetness level, and rainfall event prior to the surveys with the aim of developing more robust guidelines on the optimal timing for subsurface drainage mapping. More specifically, concerning the UAV imagery, this will be achieved by visiting a number of sites with different soil types multiple times both within and outside the growing season, as proposed by Allred et al. [40]. In relation to GPR, this could be achieved by predicting its suitability by using electromagnetic simulation software such as gprMax [80] for various drainage pipe depths and diameters to pre-emptively decide whether GPR technology is appropriate for drain line mapping at a particular site, as earlier proposed by Koganti et al. [31].

## 5. Conclusions

UAV imagery proved to be a feasible and cost-effective solution for subsurface drainage mapping as large agricultural areas can be surveyed in a limited time. However, its inability to find the linear patterns (e.g., drainage pipes) under certain circumstances and lack of distinction with other similar linear features (e.g., harvest or tillage tracks) remain caveats for this technique. While GPR was proven effective in previous studies, the technique can be inefficient and cost-intensive to cover large farm field areas and has limited applicability in high-EC soils. Thereby, given the constraints, when used appropriately, collecting GPR data along a limited spatial extent in combination with UAV imagery not only provided the depth information of the drainage pipes but was also helpful to set apart the linear signatures caused by drain lines from those caused due to field operations. At the study sites, where the UAV imagery was unsuccessful or only partly successful, a few parallel GPR transects along the edge and in the center of the field, randomly fashioned transects, and the use of spiral and serpentine transects assisted in mapping the drain lines’ location, to determine their orientation, and to guesstimate the drainage network pattern. Moreover, this was particularly helpful to know for certain if the field is subsurface drained or not, which by itself is a very important piece of information for hydrological modeling. In this relation, GPR provided complementary information and proved suitable both as a mapping and validation technique. Hence, the use of UAV imagery in combination with GPR across limited transects proved to be a more optimal approach for subsurface drainage mapping.

## Data Availability

Data available on request to the corresponding author.

## Supplementary Materials

The following are available online at https://www.mdpi.com/article/10.3390/s21082800/s1, Figure S1: GPR profile from the survey transect overlying the railway crossing (from Site-4).
