# Supplementary material for: Mapping of Agricultural Subsurface Drainage Systems Using Unmanned Aerial Vehicle Imagery and Ground Penetrating Radar
Source: Sensors (Basel). 2021 Apr 15;21(8):2800. doi: 10.3390/s21082800 (PMC8071494; doi:10.3390/s21082800)
Supplement: Supplementary file 1 [file sensors-21-02800-s001.zip › sensors-1182289-supplementary.pdf]

sensors

MDPI

Supplementary Material

Mapping of Agricultural Subsurface Drainage Systems Using Unmanned Aerial Vehicle Imagery and Ground Penetrating Radar †

Triven Koganti [1,*], Ehsan Ghane [2], Luis Rene Martinez [3], Bo V. Iversen [1] and Barry J. Allred [3]

[1] Department of Agroecology, Aarhus University, Blichers Allé 20, Tjele 8830, Denmark; bo.v.iversen@agro.au.dk
[2] Department of Biosystems and Agricultural Engineering, Michigan State University, East Lansing, Michigan 48824, USA; ghane@msu.edu
[3] USDA/ARS Soil Drainage Research Unit, 590 Woody Hayes Drive, Columbus, Ohio 43210, USA; luis.martinez@usda.gov (L.R.M.); barry.allred@ars.usda.gov (B.J.A.)
* Correspondence: triven.koganti@agro.au.dk; Tel.: +45 91732010

† This paper is an extended version of the conference paper: Koganti, T.; Ghane, E.; Martinez, L.R.; Iversen, B.V.; Allred, B.J. Mapping Subsurface Drainage in Agricultural Areas Using Unmanned Aerial Vehicle Imagery and Ground Penetrating Radar. In Proceedings of the 1st Indian Near Surface Geophysics Conference & Exhibition, New Delhi, India, 28–29 November 2019; pp. 76–80.

Citation: Koganti, T.; Ghane, E.; Martinez, L.R.; Iversen, B.V.; Allred, B.J. Mapping of Agricultural Subsurface Drainage Systems Using Unmanned Aerial Vehicle Imagery and Ground Penetrating Radar †. *Sensors* **2021**, *21*, x. https://doi.org/10.3390/xxxxx

Academic Editor: Firstname Lastname

Received: date
Accepted: date
Published: date

Publisher's Note: MDPI stays neutral with regard to jurisdictional claims in published maps and institutional affiliations.

Copyright: © 2021 by the authors. Submitted for possible open access publication under the terms and conditions of the Creative Commons Attribution (CC BY) license (http://creativecommons.org/licenses/by/4.0/).

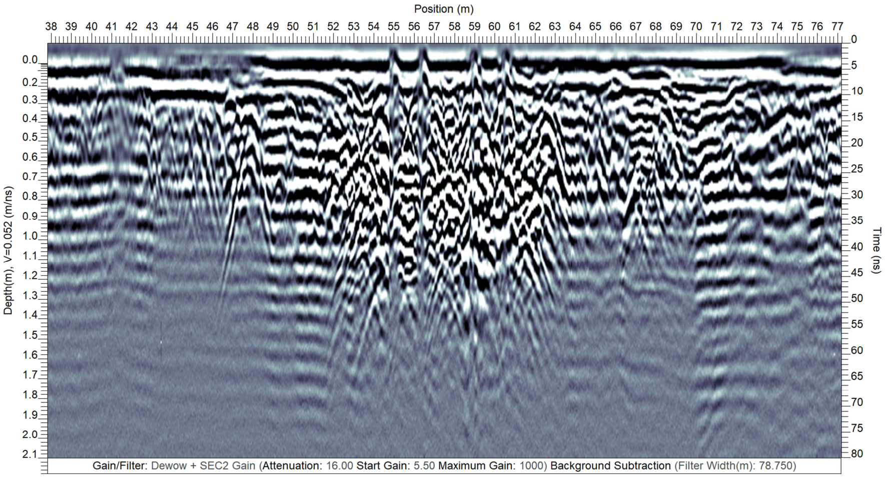

Figure S1. GPR profile from the survey transect overlying the railway crossing at Site-4.
